# Prevalence and Management of Burnout Among Dental Professionals Before, During, and After the COVID-19 Pandemic: A Systematic Review

**DOI:** 10.3390/healthcare12232366

**Published:** 2024-11-26

**Authors:** Marius Negucioiu, Smaranda Buduru, Simina Ghiz, Andreea Kui, Sebastian Șoicu, Rareș Buduru, Sorina Sava

**Affiliations:** 1 Prosthetic Dentistry and Dental Materials Department, Iuliu Hatieganu Medicine and Pharmacy University of Cluj-Napoca, 400012 Cluj-Napoca, Romaniasavasorina@elearn.umfcluj.ro (S.S.); 2 Cluj County Emergency Clinical Hospital, 400006 Cluj-Napoca, Romania; 3 Dental House Pitești Private Practice, Argeș, 110177 Pitești, Romania; sebsoicu@yahoo.com; 4 Stomestet Private Practice, 400394 Cluj-Napoca, Romania

**Keywords:** burnout, dentist, preventive therapy, COVID-19 pandemic, occupational health

## Abstract

Background: Burnout syndrome represents a significant challenge in healthcare, impacting dental professionals globally across all geographic, demographic, or socioeconomic backgrounds. Pervasive work-related stress and insufficient management practices highlight the urgent need for enhanced awareness and targeted interventions. Objectives: This systematic review aimed to evaluate the prevalence, causes, and impacts of burnout among dental professionals and to explore the effectiveness of various occupational health interventions aimed at managing and preventing burnout in the dental sector during different phases of the COVID-19 pandemic. Methods: We conducted a systematic review following the PRISMA guidelines, searching electronic databases, including PubMed, Scopus, and Web of Science, from January 2014 to April 2024. The eligibility criteria included studies reporting on the prevalence of burnout, interventions to reduce burnout, and the impact of burnout on dental practices. A quality assessment was performed using the Newcastle–Ottawa Scale for observational studies. Results: The findings indicate high levels of emotional exhaustion, depersonalization, and reduced personal accomplishment among dental practitioners, with significant variations influenced by workplace factors, professional relationships, and pandemic-related stresses. Differential effects based on educational level and age were also evident. Conclusions: This review highlights the urgent need for targeted public health initiatives and occupational health strategies to address and mitigate burnout in dentistry. Emphasizing professional relationships and workplace dynamics could play a crucial role in the prevention and management of burnout (PROSPERO registration no. CRD42024586616).

## 1. Introduction

Burnout syndrome (SBO) is defined as an excessive stress reaction caused by the workplace, leading to both physical and mental exhaustion. It is essential to emphasize that this aspect arises because of professional burnout. The individual in question begins by experiencing fatigue and tiredness, followed by a loss of interest in work and negativity related to achieved results [1].

The term “burnout” was first analyzed and defined by psychologist Herbert Freudenberger. In 1976, Christina Maslach described burnout as “emotional exhaustion, depersonalization, and reduced sense of professional accomplishment” (2). She also asserted that burnout differs from depression, although the two concepts share similarities. Alongside Susan Jackson, Maslach developed a questionnaire to assess the discussed syndrome: the Maslach Burnout Inventory (MBI). The questionnaire is a 22-item instrument that assesses burnout across three dimensions (emotional exhaustion/EE, depersonalization/DP, and personal accomplishment/PA) [2]. Although burnout affects an individual’s health, the World Health Organization (WHO) classified this syndrome as a medical condition or psychological impairment only starting in 2019 [3]. The WHO considers burnout strictly related to occupational contexts and not applicable to other personal experiences [4].

Since burnout syndrome was not initially considered a distinct medical condition, the concept of diagnostic criteria is still somewhat misunderstood. Burnout is characterized by assessing the severity of symptoms based on exhaustion, cynicism, and reduced effectiveness at work [5]. The percentage of affected individuals continues to rise. Among the most affected are young professionals who have recently entered the workforce. They exhibit a higher degree of professional involvement, often unaware of their own limits. Due to the multiple tasks young practitioners face, combined with their lack of experience, they are more susceptible to workplace stress and failure.

Regarding the level of education, it has been observed that individuals with higher education are more susceptible to burnout. This is because they often have greater responsibilities, leading to increased stress. Additionally, they may have higher expectations when it comes to achieving results. Burnout can affect anyone, but those with more demanding roles or higher educational qualifications may experience it more intensely [6]. To analyze the presence of burnout syndrome within universities, a new version of the Maslach Burnout Inventory (MBI) has been introduced and developed specifically for students. This questionnaire assesses several dimensions related to burnout, including reduced academic performance, decreased efficiency, increased workload, limited adaptability, and suicidal ideation [7].

Professions involving direct interaction with people also carry a high risk of burnout. Therefore, practitioners in medical fields are twice as likely to develop burnout compared to those in non-medical domains [8]. Physicians are influenced by the suffering of the patients they interact with every day. As patients age, treating them becomes increasingly challenging due to the multitude of medications they consume for health issues accumulated over their lifetimes. An additional stressor arises when patients fail to follow their doctor’s advice, leading to non-compliance and lack of respect [9].

In this field, it is crucial to prioritize the needs of others, work long hours, and do everything possible to assist a patient. Often, the standards for results are very high in relation to insufficient resources and income. Additionally, in the realm of medicine, there is a fear of malpractice. Burnout leads to medical errors and creates a less secure hospital environment for patients. Among the most common medical errors are judgment errors, misdiagnoses, and technical mistakes [10].

Burnout syndrome affected approximately 22.2% to 55% of dental professionals in 2019 [3] compared to 26% in 2014 [11]. The pandemic induced by the SARS-CoV-2 virus has also had an adverse impact on the mental health of medical personnel. Healthcare workers have been particularly affected by limited resources within medical institutions, limited knowledge related to the infection caused by the new virus, direct exposure to infected individuals, additional working hours, neglect of personal lives, and a lack of communication and information [7].

In a study published in 2022, the investigators showed that the overall prevalence of burnout was 13% and that emotional exhaustion is the most affected subscale. Additionally, the study highlighted that 25% of dentists experienced high EE, 18% had high DP, and 32% had low PA. The searches were conducted until January 27 of the year 2021. This study also used the Maslach Burnout Inventory questionnaire for the diagnosis of the syndrome [12]. Another study, published in 2023, investigated the prevalence of the syndrome among dentists and indicated that burnout was more common in Europe and less common in America. In addition, the authors emphasize the importance of paying attention to the mental health of dentists to avoid professional burnout [13].

Although burnout has been widely studied in the healthcare field, dental professionals represent a unique case deserving special attention, particularly in light of the COVID-19 epidemic. Previous research has tended to focus on general healthcare workers, with less attention paid to how dental professionals cope with the stressors specific to their field. It is important to note that dentists face unique challenges, such as close proximity to patients during treatment and high workloads. In addition, the existing literature often treats dental professionals as a monolithic group, neglecting the diverse experiences of dental students, general dentists, specialist dentists, and dental assistants. Our systematic review aims to fill these gaps by considering the temporal phases of pre-pandemic, pandemic, and post-pandemic recovery and by providing a comprehensive analysis of burnout across these diverse roles within dental settings.

The aims of this systematic review were to evaluate the prevalence, causes, and impacts of burnout among dental professionals and to explore the effectiveness of various occupational health interventions aimed at managing and preventing burnout in the dental sector during different phases of the COVID-19 pandemic.

This study distinctively examines how the COVID-19 pandemic has affected occupational stress and burnout among dental professionals. By synthesizing recent research, our systematic review assesses burnout’s prevalence, causes, and effects, emphasizing changes during the pandemic. It offers new insights into adapting occupational health strategies and mental health supports specifically for dental settings. These findings aim to inform policies and practices that enhance the well-being of dental professionals and improve patient care.

## 2. Materials and Methods

### 2.1. Systematic Review Design

This study adhered to the PRISMA 2020 guidelines for systematic reviews [14]. We conducted a systematic review to assess the prevalence and impact of burnout syndrome among dental personnel, including dental students, residents, specialized and non-specialized dentists, and dental assistants. This review adheres to the PRISMA-ScR (Preferred Reporting Items for Systematic Reviews and Meta-Analyses for Scoping Reviews) guidelines. A protocol was registered at PROSPERO (CRD42024586616).

This review was designed to answer the question: How has the COVID-19 pandemic affected the prevalence and management of burnout among various dental professionals, and what interventions have been effective in managing this condition?

### 2.2. Search Strategy

An electronic search strategy was developed and conducted independently by M.N and S.G in three databases: PubMed, Scopus, and Web of Science. This included identifying relevant search terms, keyword-related term branching, exploratory literature searches, database-controlled vocabulary translation, and accommodating searching particularities of each database. The search was restricted (1 January 2014–1 April 2024) to find the most recent articles available and to ensure information actuality. The process was reiterated on 15 May 2024 to discover new potentially relevant articles. Initial search terms included “burnout”, “syndrome”, “in”, and “dentistry”. To refine the search, “dentistry” was later replaced with “dentists”, and “syndrome” was omitted, leading to the final search keywords: “burnout” and “dentists”. Afterward, the strategy was developed to maximize the retrieval of relevant studies addressing burnout specifically in dental professionals.

The exact terminology used in PubMed was (“burnout” [tw] OR “professional burnout” [tw] OR “occupational burnout” [tw] OR “burnout syndrome” [tw] OR “burnout” [MeSH Terms]) AND (“dental professionals” [tw] OR “dentists” [tw] OR “dental students” [tw] OR “dental specialists” [tw] OR “dental assistants” [tw] OR “dentistry” [tw] OR “dentists” [MeSH Terms] OR “dental staff” [tw]) AND (“COVID-19” [tw] OR “SARS-CoV-2” [tw] OR “COVID-19 pandemic” [tw] OR “coronavirus” [tw] OR “COVID-19” [MeSH Terms] OR “pandemic” [MeSH Terms]).

### 2.3. Selection Criteria

The inclusion criteria were structured according to the PECOS strategy:

P (Population) = dental professionals including dentists, dental assistants, dental hygienists, and dental students.

E (Exposure) = exposure to the professional dental environment during the different phases of the COVID-19 pandemic.

C (Comparator) = pre-pandemic period or different phases of the pandemic as comparators to assess changes over time.

O (Outcome Measures) = prevalence of burnout, its impact on mental and physical health, and the effectiveness of various interventions aimed at reducing burnout.

S (Study Types) = observational studies, cohort studies, cross-sectional studies, and interventional studies. Both retrospective and prospective studies were included.

As additional criteria, we included studies published in English from January 2014 to April 2024 to cover the periods before, during, and after the COVID-19 pandemic as it relates to the emergence and response to the pandemic.

Exclusion Criteria Applied:

Studies focusing solely on non-dental healthcare professionals.

Studies not using validated instruments like the Maslach Burnout Inventory (MBI) to measure burnout.

Literature reviews, meta-analyses, editorials, and conference abstracts.

Studies unavailable in full text and studies with incomplete data on burnout prevalence or management.

### 2.4. Study Selection Process

The results of the electronic literature searches were exported from each database in library form and were subsequently imported into a reference manager (Mendeley Version 2.116.0). The duplicates were identified and removed using the same software, and a second de-duplication tool (Rayyan) was used to confirm de-duplication results [14]. The remaining articles were screened by title and/or abstract by two independent contributors to the article (MN and SG) and selected based on inclusion and exclusion criteria. Studies considered relevant to this study were manually retrieved full-text and read by 2 independent researchers. The inclusion and exclusion criteria were subsequently applied to select a final number of studies to be included.

### 2.5. Data Extraction

The following data were extracted from the included studies:

Study characteristics: authors, year of publication, title of the article, and source/journal.

Study design: the type of study (e.g., cross-sectional, longitudinal, observational, experimental).

Country/region: to assess geographic variability and applicability.

Population: characteristics of the study participants (e.g., dentists, dental hygienists, dental students).

Sample size: number of participants in the study.

Inclusion/exclusion criteria: criteria that defined how participants were selected or excluded.

Age/gender: to assess age/gender variability and applicability.

Professional demographics: years of experience, specialization, and work setting (private practice, hospital, or academic).

Measurement tools for burnout: What instruments were used to measure burnout? (e.g., Maslach Burnout Inventory, Copenhagen Burnout Inventory).

Burnout dimensions assessed: different aspects of burnout measured (e.g., emotional exhaustion, depersonalization, and personal accomplishment), frequency, and severity: How often and how severe were the burnout symptoms among the participants?

Main outcomes: primary and secondary outcomes regarding burnout.

Significant findings: summary of the most important results related to burnout, including statistical significance, effect sizes, and confidence intervals and any other relevant findings, such as impacts on professional performance, mental health, physical health, job satisfaction, etc.

Funding sources: information on who funded the study, which can indicate potential conflicts of interest; conflicts of interest: any declared conflicts of interest that could impact the study’s design, execution, or interpretation.

### 2.6. Risk of Bias and Quality Assessment

The risk of bias was quantified and assessed by two independent researchers. If any disagreement occurred, a third researcher was asked to intervene. Quality assessment of the included studies was performed using the NIH’s Quality Assessment Tool, which evaluates critical aspects such as study population, comparability of cohorts, and outcome measurement accuracy. Each study was rated according to predetermined criteria, ensuring a standardized approach to evaluating methodological rigor [15].

## 3. Results

### 3.1. Studies Characteristics

**Figure 1:**
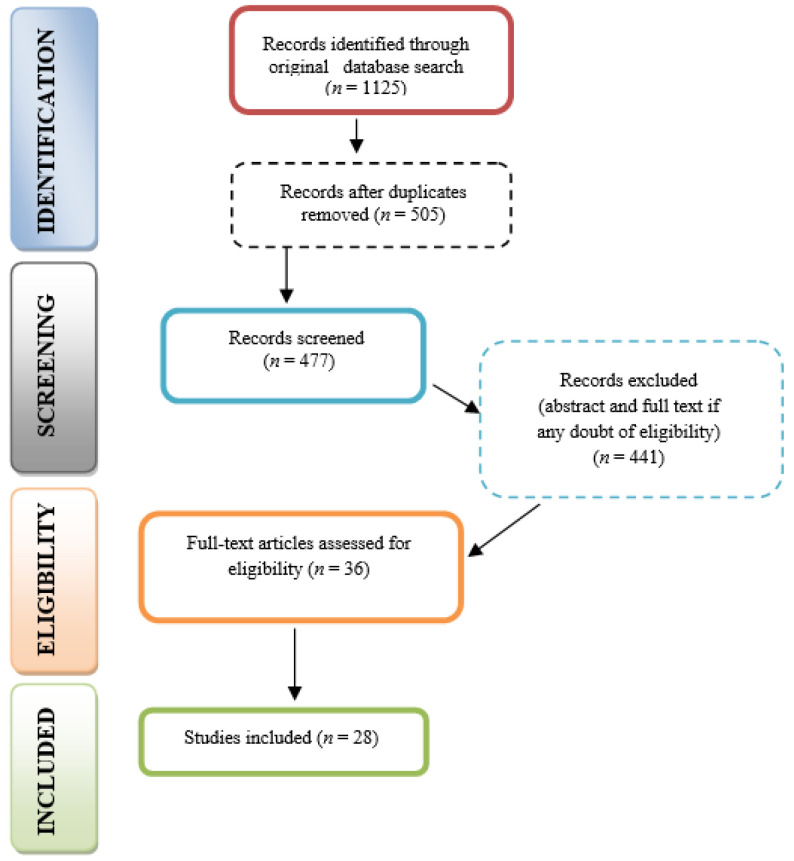
Prisma flowchart of study selection.

The search yielded a total of 1125 articles (254 from PubMed and 871 from ScienceDirect). Duplicate removal and initial screening based on titles and abstracts reduced this number to 505 relevant articles. Further screening based on full-text analysis excluded an additional 477 articles for not meeting the inclusion criteria or for lack of comprehensive data on burnout, leaving 28 articles for in-depth review (Figure 1).

The overall number of participants across all selected studies was 10,226, with 6205 women, 4009 men, and other 12 individuals who did not want to identify with traditional gender labels.

The articles included in this review (Table 1) are cross-sectional surveys that used questionnaires distributed through various online platforms (email, WhatsApp, Facebook, Instagram, Google Forms) [16,17,18,19,20,21,22,23,24,25,26,27], during conferences [19,28], via post services distribution [29], and in-person interactions [21,25,30,31,32,33]. Participants signed an informed consent form and remained anonymous.

### 3.2. Burnout Measure

These studies aimed to explore the correlation between burnout, stress, and/or depression using different types of questionnaires [16,17,20,21,22,23,24,25,26,27,31,32,33,35,37]. Notably, The Maslach Burnout Inventory–Human Services Survey (MBI-HSS) remains the gold standard for assessing burnout syndrome (Table 2). Compared to other burnout measures, the MBI has undergone more psychometric study, and because of its multidimensional definition of burnout, it is particularly well-suited for theory-driven research [2].

### 3.3. Sociodemographic Aspects and COVID-19 Era

Among the articles used for this study, eight evaluated how the COVID-19 pandemic induced the occurrence of burnout syndrome among dentists, nurses, or students or how it modified the syndrome that was already present in various countries (Brazil, Greece, Canada, Pakistan, Scotland, Turkey and Republic of Korea) (Table 3). The other articles identified and analyzed personal and workplace-related factors, as well as lifestyle aspects that contribute to the development of the syndrome, including its correlation with the blood type of the physician [35].

Sociodemographic aspects and work-related variables that have been evaluated are sex, age, marital status, skin color, monthly family income, amount of time worked as a dentist, employer economic sector (public, private, or both) [24], setting in which they practice (rural/urban), frequency with which they work alone, whether they work in several practices, number of hours they work per week, years of work experience, whether they own a practice, greatest commute time to place of work, satisfaction with salary (1), number of children, number of patients seen per day [41], specialty, workload, and work hazards [21].

### 3.4. Comparison Between Dentists

Additionally, the presence of the syndrome was analyzed in dental students [29,32,34,35,36,37,38]. Levels of emotional exhaustion (EE), depersonalization (DP), and personal accomplishment (PA) were compared among general dentists and dentists with various specializations (orthodontists, pediatric dentists, periodontists, maxillofacial surgeons, implantologists, prosthodontists, estheticians, endodontists, and dental hygienists) [17,18,21,25,27,30], as well as dental nurses [33].

Some studies support the presence of burnout among students at a rate of 17.8% [32,36], 28.8% [37], and during the COVID-19 pandemic at a rate of 32% [34]. Burnout is more pronounced in those who are taking their first steps in their professional field. Among students with present burnout, the majority have blood type B II [35]. Compared to male dental students, female students experienced higher levels of stress. Burnout is linked to stress. These results corroborate the notion that female dentists experience higher levels of burnout [29,35]. Republic of Korean students expressed low PA scores [36].

Different specialties such as oral surgery and implantology exhibit a higher level of burnout [30,41]. Interventions involving significant blood can scare patients, thereby stressing the specialist physician. Over time, the level of depersonalization among physicians increases. Also, periodontists (degree of burnout of 13.6%) can experience an increased level of emotional exhaustion for the same reasons as surgeons [17]. The percentage of American pediatric dentists who have burnout is 9.1% for the test with dual criteria (high EE and high DP) and 2.2% for all three criteria (high EE, high DP, and low PA) [27].

### 3.5. Presence of Burnout Syndrome and Distribution of Burnout Subscales

In 15 of the articles included in this review, the presence of burnout syndrome was highlighted in percentage terms (Table 4).

The highest level of burnout was recorded in Lithuania (44.49%) [28].

Some of the articles included in this review (Table 4) presented the distribution of burnout subscales based on geographical location and the publication year [1,16,17,19,20,21,22,24,26,27,28,29,30,31,32,33,34,35,36,37,38,39,40,41].

The highest level of emotional exhaustion (EE) was identified in Turkey (91.72%) [35], depersonalization (DP) in the Republic of Korea (90.40%) [37], and the lowest level of personal accomplishment (PA) in Thailand (4%) [20].

### 3.6. Risk of Bias/Quality Assessment of Studies

The quality assessment of the studies included revealed a general trend of moderate to high quality across the research (Table 5), with significant variability in methodological rigor. Many of the included studies clearly stated their objectives and defined outcome measures, yet some lacked specified eligibility criteria and comprehensive controls for confounding variables. Notably, some studies did not employ appropriate statistical analysis or offer thorough reporting, which may introduce biases and limit the reliability and generalizability of the findings. These methodological shortcomings underscore the need for more robust study designs and detailed reporting in future research on burnout among dental professionals.

## 4. Discussion

Burnout syndrome is a significant issue affecting professionals across various fields, including healthcare. Through this study, the level of impact on personnel from various branches of dentistry by occupational burnout is highlighted.

Additionally, within the selected articles, several factors leading to the development of this syndrome are identified, along with the cognitive and behavioral efforts that employees make to cope with increasing demands. It is evident that dentists can easily develop professional burnout regardless of their geographical location or their area of expertise within the field of dentistry.

The decision to focus our search starting with the year 2014 was made based on several factors that ensure our study’s relevance and rigor. Firstly, the modern dental practice landscape has evolved significantly due to advances in dental technology, changing practice standards, and new regulatory environments, which are best reflected in the most recent data. The period since 2014 has also seen a surge in high-quality research into dental burnout. This provides a robust dataset that captures the nuances of current occupational health challenges. In addition, this time period coincides with major global health and economic events, such as the Ebola outbreak and the aftermath of the 2008 financial crisis, which have had an impact on healthcare practices and their impact on burnout levels. Limiting the review to the last ten years allows for a manageable analysis of the data, ensuring a thorough and focused examination of the changes in burnout prior to the unique stresses of the COVID-19 pandemic, thereby providing a clear understanding of the underlying trends in the occupational health of dental professionals.

This review’s findings reveal that EE is directly affected by environment, age, gender, working hours per week, low job satisfaction, working on weekends, and a high number of patients [1,19,20,27,30], DP by ownership of the practice, years of experience, age, specialists, and working hours per week, and PA by working alone, never being married, and having a teaching position [1,19,20,29,39]. Also, personality factors such as neuroticism, extraversion, agreeableness, and conscientiousness can predict burnout syndrome [16].

Young dentists, who work long hours every week without possessing private practice, are more likely to experience burnout syndrome [1]. Dentists who work overnight have a higher EE level than other dentists [29]. Compared to general practice dentists, specialized dentists were especially less likely to have burnout syndrome [18,31].

Private dentists who worked in residential areas had higher mean DP scores compared with those who practiced in business settings [31,38]. The aspects of depersonalization and lack of personal accomplishment were directly linked to cigarette smoking [28,41].

Compared to other studies, this study included articles about burnout syndrome in dentistry during the COVID-19 pandemic.

Through studies conducted during the COVID-19 pandemic or for assessing how the pandemic has affected the level of burnout, several aspects have been observed. Factors associated with the development of burnout during the pandemic include fear of COVID-19 infection, increased workload due to the pandemic, practice put on hold because of the pandemic [24], loss of income resulting from further lockdowns, the cost of personal protective equipment, patient volume decline [25], and handling patient’s concerns while providing essential dental care [40].

All three components of burnout syndrome were associated with a high degree of anxiety, increased alcohol consumption, less time allocated to work, and assistance from colleagues [24]. Younger employees, with fewer years of experience, were more predisposed to developing the syndrome. Elderly dentists appear to have internal resources that enable them to overcome anxiety and financial hardship [25]. Additionally, participants who wore N95 masks during clinical procedures experienced twice as much stress and burnout compared to those who wore FFP3 masks [34]. Students practicing in a clinical environment also exhibited higher levels of EE and DP because they needed to recruit and perform dental treatment for patients [37].

A significant difference compared to the pre-pandemic period is that men are more predisposed to emotional exhaustion (EE) than women [25], but this is not the case in Romania, where women have shown higher levels of EE and DP [26]. Additionally, fatigue and EE were much easier to develop during the pandemic [25]. Dentists who worked more than 16 h per week were more prone to EE, and those who worked more than 40 h had better DP outcomes [22]. Among patients with high EE levels, there was an expressed intention to change their workplace. Career decisions may be significantly influenced by burnout, especially for people with greater levels of EE and DP. Women are more likely to change their profession [26].

Lower levels of emotional exhaustion (EE) and depersonalization (DP) have been associated with a sense of safety among personnel when using protective equipment [24]. It has been observed that dentists with a postgraduate degree are less affected by burnout syndrome due to greater enumeration. A higher educational level seems to be a protective factor against exhaustion, possibly because dentists can find more inner worth and self-compliance than others [23,38].

It is essential that dentists keep up with the latest developments in dentistry to handle growing demands and difficulties from patients and society. A good relationship between dentists and assistants can prevent a high EE degree on dentist performance [21]. An optimal work environment, marriage, pregnancy, and work–life balance determines less burnout among professionals [17]. The most popular methods of relaxation to reduce the level of burnout seem to be spending time with family and friends, sleeping, eating and cooking, listening to music, watching TV, playing sports, and taking time to enjoy nature [28].

The study titled “Systematic review: factors contributing to burnout in dentistry” published in 2015 identified factors associated with the syndrome: younger age, male gender, student status, high job-strain/working hours, and certain personality types [43]. Since 2015, further studies have been conducted, and other factors have been discovered. Thus, it was observed that women are more affected by EE and men by DP [18,25,26,32,36].

Plessas et al. investigated the optimal measures to prevent, improve, or tackle mental health issues among dental specialists. This systematic review identified articles only about dentists and dental students, while our study identified articles about other sectors as assistants or professors in dental fields [44].

Certain articles have emphasized that a simple two-item burnout screening tool based on the MBI can be used to identify a possible burnout syndrome but also to raise awareness [45].

Our review, along with all other systematic reviews, underscores the critical significance of mental health and the prevention of burnout syndrome. Professional burnout impacts every facet of life, encompassing personal well-being, and it also influences the oral health outcomes of patients. The consequences of this syndrome end up affecting both the doctor and the patient. Problem solutions will be inadequate or cease to appear, thus decreasing work quality. Within the medical field, the patient becomes a permanent stressor for the practitioner. Repeated poor results will lead the employees to consider themselves professionally incompetent.

Emotional exhaustion, multiple responsibilities at work, exposure to stress-causing factors, personal vulnerability, sleep deprivation, and lack of rest can lead to the adoption of coping strategies. The individual in question may resort to consuming various substances to reduce stress levels, substances that can become addictive [24,28,30].

Burnout leads to medical errors and a less secure hospital environment for patients. Among the most common medical errors are judgment errors, misdiagnoses, and technical mistakes. Excessive stress to which doctors are subjected daily leads to an illusion of increased efficiency, but over time, it results in chronic fatigue. Although stress can sometimes have a positive or productive effect (known as eustress), when adapted as a working style, it leads to negative effects for both the employee and the outcomes [46,47].

It is critical that dentists feel positive about their work environment because this will lessen burnout episodes and increase job satisfaction [17]. It is recommended that dentists stay up to date on their knowledge and abilities to prepare them for the growing demands and difficulties that society and patients are posing [38]. It is suggested that dental education programs be designed to start stress management and mitigation training at the beginning of the dental program to encourage students to develop healthy coping mechanisms for the future. Using these techniques over the course of their careers will help them stay in the field and provide the best possible care for their clients [22,26].

### 4.1. Theoretical Implications

By analyzing how burnout manifested and was managed in this specific health sector, our study contributes several important theoretical insights that can inform future research and practice. First, our study reinforces and extends the theoretical framework of the Maslach Burnout Inventory (MBI) by contextualizing its dimensions (EE, DP, PA) in a pandemic setting. The significant increases in emotional exhaustion and depersonalization reported highlight the acute impact of pandemic-related stressors, such as increased work demands and health risks. These findings suggest that the theoretical model might need to be adapted to include external systemic stressors such as pandemics.

Regarding the impact of organizational support systems, our findings suggest that proactive strategies, such as the implementation of structured wellness programs and ergonomic improvements, are critical in preventing and reducing burnout. This study also highlights the importance of interdisciplinary approaches in addressing occupational burnout. The interaction between psychological, organizational, and physical health factors suggests that burnout theories could benefit from incorporating insights from public health, occupational behavior, and ergonomics. This interdisciplinary perspective could enrich the theoretical understanding of burnout by illustrating how multifaceted interventions can address the complex nature of burnout syndromes.

Finally, our findings suggest that theories of occupational stress and burnout need to consider the dynamic nature of healthcare environments, where rapid changes (such as those induced by a pandemic) require equally dynamic responses. This calls for an extension of existing theories to include concepts of adaptability and resilience, not only at the individual level but also at the organizational and systemic levels.

### 4.2. Limitations

The limitations of our systematic review refers to several aspects, such as the following: (1) limitations of single surveys: Different survey methods to assess burnout means were identified among the studied included, which may limit the ability to fully understand the complex nature of burnout and its causes, particularly in relation to interventions that were not consistently evaluated across the included studies; (2) variability between countries: The incidence and perception of burnout can vary widely in different national contexts, influenced by different healthcare systems and organizational cultures; (3) inconsistency in measurement: The use of the Maslach Burnout Inventory (MBI) across studies led to heterogeneity in the data, as the use of the MBI and the interpretation of its severity scales varied widely, which could affect the reliability of comparisons between studies, especially when aggregating data to identify global trends; (4) single point assessments: Many studies have assessed burnout at a single point in time rather than longitudinally; (5) response bias: Not all dental professionals surveyed responded to the surveys, and those who did may represent a group more likely to report problems due to personal or contextual factors, such as higher levels of stress or dissatisfaction with their work environment; and (6) limited evaluation of interventions: Although some interventions were highlighted as effective in managing or reducing burnout, this review could not always determine the long-term effectiveness of these interventions; also, the lack of randomized controlled trials focusing on the effectiveness of interventions insufficiently supports specific burnout management strategies among dental professionals.

### 4.3. Practical Implications

This systematic review highlights the critical need for comprehensive occupational health promotion strategies and effective management of burnout in the dental profession. Our findings reveal a significant prevalence of burnout. This negatively impacts the well-being and operational efficiency of dental professionals. To address this, we propose several specific interventions tailored to the unique dental environment:

Structured wellness programs: We recommend the implementation of structured wellness care that proactively addresses mental health. These could include regular mental health assessments and targeted stress management but also coping skills workshops, mindfulness sessions, and access to psychological counseling.

Ergonomic interventions: To reduce emotional exhaustion, an important dimension of burnout, dental practices should implement comprehensive ergonomic interventions. These include training in good ergonomic practices, providing supportive seating options, optimizing the design of dental tools, and structuring work schedules to include regular breaks. Such measures would help reduce physical stress and mental fatigue among dental professionals.

Team-based care models: To distribute the workload more evenly among staff, the implementation of a team-based care approach is essential. As well as reducing the pressure on individual professionals, this model promotes a collaborative working environment that may increase job satisfaction and personal performance. A collaborative and supportive workplace culture can be reinforced through regular team meetings and shared responsibilities.

Professional development and burnout education: Mandatory continuing education programs on burnout recognition and management are essential. Such training would help dental professionals recognize early signs of burnout in themselves and colleagues and implement effective, evidence-based interventions.

Implement mental health support policies: Dental institutions need to establish and enforce policies that actively promote mental health. It is recommended that policies ensure mandatory time off, provide support for mental health issues, and create an open environment where mental health issues can be discussed freely and without stigma.

Monitoring and feedback mechanisms: To ensure the effectiveness of these policies, regular monitoring and adaptive feedback mechanisms need to be in place. The implementation of anonymous surveys and feedback tools will help to continuously assess levels of burnout and the success of ongoing interventions, allowing for timely adjustments.

Preparing for future pandemics: The COVID-19 pandemic demonstrated the need for adaptable strategies that can respond to increased stressors during global crises. Future contingency planning should include protocols for maintaining mental health support and rapidly adapting work practices to effectively manage unexpected stressors.

## 5. Conclusions

This systematic review underscores the escalating prevalence of burnout syndrome among dental professionals, ranging from students to seasoned practitioners. The results emphasize the critical need to recognize the early signs of burnout, which are pervasive across all levels of dental practice. Prompt identification and management are essential to prevent the progression of more severe mental health conditions such as depression. The Maslach Burnout Inventory (MBI) continues to serve as the gold standard for assessing burnout, providing a reliable tool for diagnosing and measuring its severity within the dental community.

The COVID-19 pandemic has notably exacerbated burnout syndrome, intensifying stress and anxiety among dental professionals and thereby impacting their career trajectories. This phenomenon highlights the necessity for robust clinical strategies aimed at mitigating burnout. Implementing structured wellness programs, ergonomic adjustments, team-based care models, and professional education about burnout are vital steps toward enhancing occupational health in dentistry. These interventions not only address the symptoms but also the underlying causes of burnout, facilitating a healthier work environment and improving both personal and professional life balance.

Moreover, the establishment of a collegial and supportive workplace culture is instrumental in preventing the onset of burnout. This environment encourages open discussions about mental health challenges and promotes preventive measures, ensuring that dental professionals can maintain a sustainable balance between their work demands and personal needs.

To advance our understanding of burnout and to refine intervention strategies, ongoing research is imperative. Future studies should focus on the long-term effects of the pandemic on dental health professionals, explore how technological advancements might alleviate or compound burnout, and identify effective measures for preventing burnout in evolving clinical settings.

In conclusion, by aligning clinical practices with the strategic management of burnout, dental professionals can enhance their occupational health, thereby ensuring their well-being and the efficacy of the dental care they provide.

## Figures and Tables

**Table 1:** Studies assessing burnout syndrome in dentistry.

Ref. No.	Aim of the Study	Scales	Conclusions
[1]	Evaluate the sociodemographic and work-related factors that influence the degree of syndrome development through an observational epidemiological study in Spain.	Maslach Burnout Inventory	EE is influenced by age and working hours per week. Ownership of the practice, years of experience, and working hours per week impact DP. Working alone determines a high risk of low PA.
[16]	To use epidemiological research to examine the personality traits and coping mechanisms used by Spanish dentists who are experiencing burnout syndrome.	MBI-HSS (burnout questionnaire) NEO-FFI (personality traits) Brief COPE (coping styles)	Strong indicators to try and predict whether dentists may have burnout syndrome are personality factors, avoidant coping style, and age.
[17]	To investigate the connections between dentists’ work environments, job satisfaction, and burnout and to investigate how particular sociodemographic factors predict how dentists perceive their work environments in Spain.	Maslach Burnout Inventory Survey of Organizational Attributes for Dental Care Warr–Cook–Wall Overall Job Satisfaction Scale	A good perception of well-being at work is perceived by dentists with more years of professional experience. Reducing burnout is important to improve well-being in dentistry.
[30]	To determine the incidence of burnout in professionals dedicated to dentistry in Spain.	Maslach Burnout Inventory	Oral surgery and implantology seem to be risk activities for the manifestation of depersonalization.
[18]	To investigate the performance of the items and the subscales of the Maslach Burnout Inventory among dental professionals in Lithuania.	Maslach Burnout Inventory	MBI offers factorial validity and demonstrates its invariant structure and variance of burnout dimensions across demographic and workload groups.
[19]	To evaluate the burnout level among Lithuanian dentists and its association with demographic variables, job satisfaction, and other job-related variables.	Maslach Burnout Inventory	In Lithuania, dentists can develop a high degree of burnout, especially high emotional exhaustion. Low job satisfaction appears to be an important factor in all three burnout dimensions.
[28]	To determine the association between burnout level with lifestyle and relaxation among Lithuanian dentists.	Maslach Burnout Inventory	Active relaxation is positively associated with burnout and an unhealthy lifestyle is negatively associated with the syndrome.
[31]	To assess the relationship between depressive symptoms and burnout among dentists in Turkey.	Maslach Burnout Inventory (MBI) Beck Depression Inventory (BDI)	A high level of burnout may provide the opportunity to identify depression earlier.
[32]	To determine the prevalence of burnout and occupational participation limitation among dental students in Turkey.	Maslach Burnout Inventory-Student Version (MBI-SV) Canadian Occupational Performance Measure (COPM)	Burnout and occupational participation limitations are present among dental students. They are both capable of existing simultaneously.
[34]	To evaluate how COVID-19 pandemic affects dentists’ competence and willingness to work, as well as its impact on occupational burnout in Turkey.	Maslach Burnout Inventory	The dentists who served during the pandemic showed higher stress and burnout levels.
[35]	To determine the professional and personal factors impacting depression and burnout syndrome in dentists working in private clinics, academic faculties, and oral and dental health centers in Turkey.	Maslach Burnout Inventory Beck Depression Inventory (BDI)	Oral and dental health center dentists reported greater levels of EE.
[29]	To determine the degree and distribution of burnout and to evaluate the factors associated with the syndrome among Republic of Korean dentists.	Maslach Burnout Inventory	High burnout level is associated with some job-related and sociodemographic factors. Burnout is a common problem in Republic of Korean dentists.
[36]	To investigate the burnout and depression levels among Republic of Korean senior dentistry students.	Maslach Burnout Inventory Patient Health Questionnaire (PHQ-9)	When compared to students who were not burnt out, burnout students reported far lower satisfaction levels and a greater need for counseling.
[37]	To assess the relationship between COVID-19 and stress, burnout, and depression among dental students in Republic of Korea.	Maslach Burnout Inventory (MBI) Patient Health Questionnaire-9 (PHQ-9) Impact of Event Scale-Revised (IES-R)	Students who work with patients experience higher levels of depression, vulnerability because of COVID-19, and burnout.
[38]	To evaluate occupational burnout and its association with stress among dentists in Hong Kong.	Maslach Burnout Inventory	A high level of burnout was associated with job-related stressors and a lack of postgraduate qualifications.
[39]	To investigate work stress and occupational burnout among dentists in Taiwan.	Maslach Burnout Inventory-Human Service Survey (MBI-HSS) The Questionnaire on Medical Workers’ Stress (QWS)	Professional stress and burnout were common among dental staff and better stress management is recommended to promote mental health.
[20]	To determine the level of burnout and related factors among dentists in southern Thailand who work in public hospitals.	Burnout Inventory–Human Services Survey (MBI-HSS) Thai Effort–Reward Imbalance Questionnaire (Thai ERIQ) Utrecht Work Engagement Scales-9 (UWES-9)	Age and work engagement are common factors associated with EE, DP, and PA.
[33]	To evaluate professional job burnout and temperament from the viewpoint of Traditional Persian Medicine.	Maslach Burnout Inventory Salmannejad Mizaj questionnaire.	A higher degree of burnout in EE is experienced by dentists with cold and dry temperaments and a higher DP and a low PA are experienced by dentists with warm and wet temperaments.
[40]	To determine the prevalence and degree of burnout during the COVID-19 pandemic in practicing Pakistani dentists.	Maslach Burnout Inventory	All three dimensions of burnout were found to be in the moderate range and measures should be taken to manage it.
[21]	To analyze occupational burnout and work stress among dental assistants in Israel and to discover the factors predicting burnout.	Maslach Burnout Inventory Work Stress Inventory (WSI)	Predictors for EE are dentist-assistant relationship, workload, patient type, and salary. DP is influenced by patient suffering, dentist-assistant relationships, years of professional experience, and work hazards.
[41]	To study the factors associated with burnout syndrome in dental specialists in Colombia.	Maslach Burnout Inventory Copenhagen Questionnaire (CQ)	The study observed that smoking is associated with burnout syndrome and that a relationship exists between the syndrome and the years after graduating from the specialty.
[22]	To examine the prevalence of EE, DP, and low PA in dental hygienists in Canada after the COVID-19 pandemic and to explore the pandemic effects on their professional lives.	Maslach Burnout Inventory Two open-ended questions	The first wave of the pandemic may have influenced the rate of EE among dental hygienists. It is important to recognize the signs and symptoms of burnout to reduce its detrimental effects.
[23]	To look into aspects of health and well-being that dentists and other dental health workers in primary dental care experience as a result of the ambiguous surroundings of the pandemic in Scotland.	Maslach Burnout Inventory for EE and DP Patient Health Questionnaire−2 (PHQ-2) Dental Professional Preparedness for Practice Scale (DPPPS) Event Scale-Revised	The results highlight the potential advantages of allocating resources for staff support and interventions to help dental personnel in times of significant uncertainty.
[24]	Identifies the factors related to burnout among dentists during the COVID-19 pandemic in Brazil.	Maslach Burnout Inventory Beck Anxiety Inventory WHOQol-BREF questionnaire	Factors such as quality of life, amount of time in the profession, use of personal protective equipment, support from fellow dentists, and alcohol consumption should be considered during a pandemic.
[25]	To analyze occupational burnout, career satisfaction, and the quality of life among dentists in Athens, Greece.	Maslach Burnout Inventory Copenhagen Questionnaire (CQ)	Males seem to be more inclined to develop dissatisfaction, physical and emotional exhaustion, and unhappiness.
[26]	To examine the relationship between burnout and dentists’ intentions to change careers during the COVID-19 pandemic in Romania.	MBI–Human Services Survey for Medical Personnel—MBI-HSS (MP)	Higher levels of EE are more evident in female dentists and DP in male dentists, and there were no differences in PA levels between the two genders. The results were alarming and emphasized the need for interventions and support services.
[42]	To evaluate burnout and work engagement among dentists in U.S.	Maslach Burnout Inventory–Human Services Survey Utrecht Work Engagement Scale	There was a substantial correlation between the burnout subscale scores and the work engagement subscale scores.
[27]	To find out how common depression and/or burnout are in the U.S. pediatric dentistry community.	Maslach Burnout Inventory Patient Health Questionnaire-8	There was noteworthy relationship found between moderate-to-severe depression and high emotional exhaustion, high depersonalization, and low personal accomplishment.

**Table 2:** Burnout Scale.

Burnout Scale	Domains	Items	Scales	Focus
The Maslach Burnout Inventory–Human Services Survey (MBI-HSS)	Emotional exhaustion/EE Depersonalization/DP Personal accomplishment/PA	22 items	7-point scale	To evaluate a physician’s level of burnout

**Table 3:** Characteristics of the included studies.

Article	Country	Year of Publication	Study Carried out About COVID-19 Era	Definition of High-Level Burnout	Response Rate, %	Mean Age	High Level of Burnout
[1]	Spain	2022	No	EE > 26 DP > 9 PA < 3	3.4%	41.9 years (SD = 11.5)	9.80%
[16]	Spain	2021	No	EE ≥ 26 DP ≥ 9 PA ≤ 34	3.4%	41.9 years (SD = 11.5)	9.80%
[17]	Spain	2021	No	EE ≥ 24 DP ≥ 9 PA ≤ 39	9.4%	37.6 years (SD = 9.6)	3.80%
[30]	Spain	2023	No	EE ≥ 27 DP ≥ 10 PA ≤ 34	-	-teachers: 39.3 years -1st year postgraduate: 25 years -2nd year postgraduate: 25.8 years -3rd year: 27.2 years	22.20%
[18]	Lithuania	2020	No	-	3.5%	37.3 years (SD = 12.9)	-
[19]	Lithuania	2021	No	EE ≥ 27 DP ≥ 13 PA ≤ 31	61.5%	37.3 years (SD = 12.9)	15.30%
[28]	Lithuania	2021	No	-	61.5%	≥30 years	44.49%
[31]	Turkey	2016	No	-	76%	36 years (SD = 4.45)	29%
[32]	Turkey	2016	No	-	-	-	26%
[34]	Turkey	2021	Yes	EE ≥ 31 DP ≥ 11 PA ≤ 35	88.25%	-	20%
[35]	Turkey	2024	No	Burnout syndrome ≥ 18 DP ≥ 10 PA ≤ 26	-	36.98 years (SD = 5.56)	-
[29]	Republic of Korea	2015	No	EE ≥ 27 DP ≥ 10 PA ≤ 33	45.9%	45.6 years	
[36]	Republic of Korea	2020	No	EE ≥ 26 DP ≥ 10 PA ≤ 33	76.9%	26.8 years	17.90%
[37]	Republic of Korea	2024	Yes	EE ≥ 27 DP ≥ 10 PA ≤ 33	-	25 years	28.80%
[38]	China	2017	No	EE ≥ 27 DP≥ 13 PA ≤ 31	28.3%	-	7%
[39]	Taiwan	2019	No	-	79.9%	-	-
[20]	Thailand	2022	No	-	56%	34.6 years (SD = 7.6)	-
[33]	Iran	2022	No	EE ≥ 27 DP ≥ 13 PA ≤ 39	82.76%	35.2 years (SD = 7.2)	-
[40]	Pakistan	2023	Yes	EE ≥ 27 DP ≥ 13 PA ≤ 39	-	-	-
[21]	Israel	2018	No	EE ≥ 31 DP ≥ 11 PA ≤ 35	46.4%	30.9 years (SD = 9.84)	-
[41]	Colombia	2022	No	EE ≥ 27 DP ≥ 10 PA ≤33	83.7%	44.0 years (SD = 7.8)	-
[22]	Canada	2021	Yes	EE ≥ 27 DP ≥ 10 PA ≤ 33	34.9%	-	36.20%
[23]	Scotland	2021	Yes	-	27%	37 years	-
[24]	Brazil	2023	Yes	EE ≥ 30 DP ≥ 12 PA ≤ 33	-	32.1 years (SD = 8.3)	-
[25]	Greece	2022	Yes	-	12.76%	-	-
[26]	Romania	2023	Yes	-	-	38.79 years	-
[42]	USA	2017	No	-	-	-	13.20%
[27]	USA	2020	No	-	11.4%	30–40 years	9.1%

**Table 4:** Distribution of burnout subscales.

Study	EE	DP	PA
[1]	61.30%	45.80%	14.60%
[16]	30.80%	10.30%	39.80%
[17]	45.60%	20.50%	96%
[30]	25%	77.80%	11.10%
[19]	42.30%	18.70%	28.20%
[28]	24.27%	7.78%	12.44%
[31]	38%	22%	12%
[32]	25%	18%	14%
[34]	20.83%	7.58%	9.41%
[35]	91.72%	66.21%	75.87%
[29]	41.20%	55.90%	41.40%
[36]	44.60%	36.60%	51.80%
[37]	86.50%	90.40%	45.50%
[38]	25.40%	17.20%	39%
[39]	26.31%	9.60%	19.23%
[20]	45.80%	44.30%	4%
[33]	8.30%	7.50%	64.20%
[40]	47.50%	23%	29.40%
[21]	26.40%	12%	27.80%
[41]	3.40%	4.30%	4.30%
[22]	65%	34%	24%
[24]	43.40%	26.30%	81%
[26]	66.66%	20.28%	46.37%%
[27]	23%	12%	10%

Emotional exhaustion (EE), depersonalization (DP), and personal accomplishment (PA).

**Table 5:** Results of the risk of bias/quality assessment.

No.	Reference	D1	D2	D3	D4	D5	D6	D7
1	Gómez-Polo, C. et al. (2022) [1]	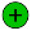	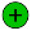	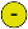	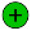	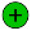	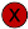	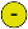
2	Gómez-Polo, C. et al. (2021) [16]	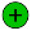	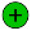	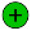	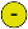	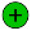	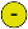	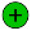
3	Molina-Hernández, J. et al. (2021) [17]	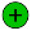	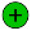	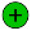	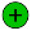	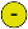	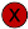	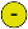
4	Rey-Martinez, et al. (2023) [30]	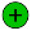	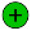	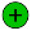	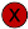	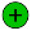	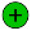	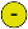
5	Slabšinskienė, E. et al. (2020) [18]	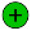	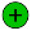	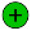	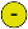	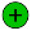	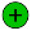	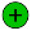
6	Slabšinskienė, E. et al. (2021) [19]	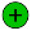	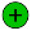	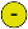	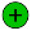	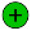	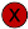	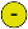
7	Slabšinskienė, E. et al. (2021) [28]	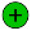	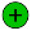	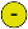	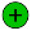	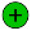	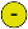	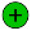
8	Huri, M. et al. (2016) [31]	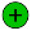	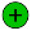	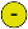	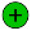	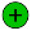	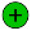	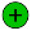
9	Eren, H. et al. (2016) [32]	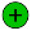	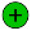	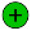	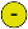	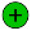	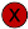	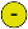
10	Özarslan, M. et al. (2021) [34]	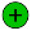	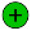	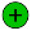	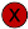	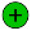	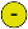	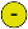
11	Ciğerim, L. et al. (2024) [35]	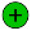	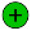	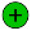	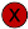	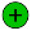	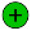	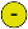
12	Jin, M.U. et al. (2015) [29]	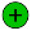	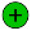	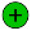	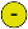	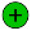	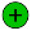	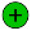
13	Kwak, E.J. et al. (2021) [36]	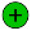	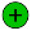	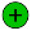	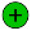	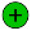	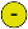	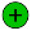
14	Kwak, G.H. et al. (2024) [37]	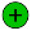	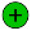	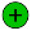	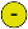	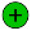	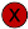	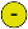
15	Choy, H.B. et al. (2017) [38]	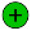	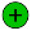	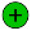	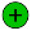	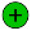	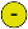	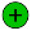
16	Lee, C.Y. et al. (2019) [39]	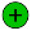	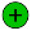	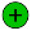	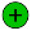	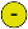	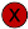	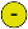
17	Na Nakorn, S. et al. (2022) [20]	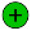	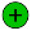	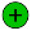	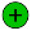	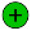	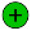	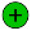
18	Noori, F. et al. (2022) [33]	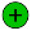	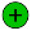	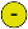	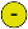	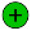	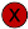	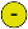
19	Ahmad, Z. et al. (2023) [40]	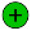	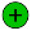	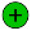	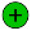	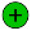	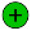	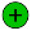
20	Uziel, N. et al. (2019) [21]	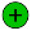	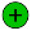	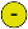	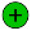	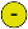	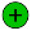	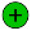
21	Hernández, S.M. et al. (2021) [41]	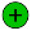	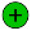	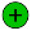	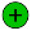	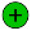	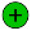	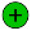
22	Haslam, S.K. et al. (2022) [22]	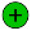	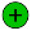	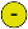	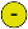	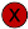	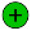	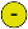
23	Humphris, G. et al. (2021) [23]	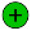	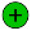	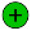	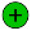	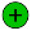	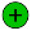	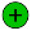
24	Silva, J.K.F. et al. (2023) [24]	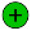	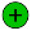	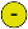	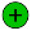	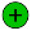	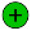	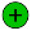
25	Antoniadou, M. et al. (2022) [25]	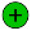	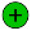	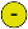	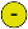	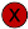	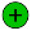	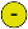
26	Silistraru, I. et al. (2023) [26]	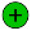	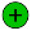	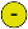	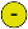	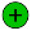	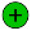	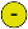
27	Calvo, J.M. et al. (2021) [42]	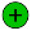	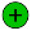	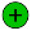	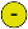	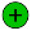	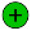	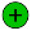
28	Chohan, L. et al. (2020) [27]	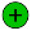	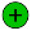	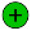	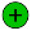	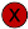	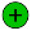	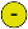

D1—Clearly stated objective; D2—Eligibility criteria specified; D3—Outcome measures clearly defined; D4—Statistical analysis appropriate; D5—Confounding variables; D6—Comprehensive reporting; D7—Overall quality (Judgment: 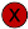
—Serious; 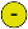
—Moderate; 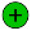
—Low).

## Data Availability

Dataset available on request from the authors.
